# Cluster headache: state of the art in treatment

**DOI:** 10.3389/fpain.2023.1265540

**Published:** 2023-10-27

**Authors:** Ildefonso Rodriguez-Leyva, Maria-Karina Velez-Jimenez, Silvia García, Juan Alberto Nader-Kawachi, Adriana Patricia Martínez-Mayorga, Agustín Melo-Carrillo, Humberto Juárez-Jimenez, Marco Martinez-Gurrola, Manuel Gudiño-Castelazo, Erwin Chiquete, Jorge Villareal-Careaga, Alejandro Marfil, Paul David Uribe-Jaimes, Rubén Dario Vargas-García, Miguel Angel Collado-Ortiz, Daniel San-Juan

**Affiliations:** ^1^ Department of Neurology, Faculty of Medicine, Central Hospital “Dr. Ignacio Morones Prieto,” Universidad Autónoma de San Luis Potosi, San Luis Potosi, Mexico; ^2^ Department of Neurology, Hospital Angeles Lomas, Mexico City, Mexico; ^3^ Clinical Research Department, Centro Médico Nacional “20 de Noviembre,” ISSSTE, Mexico City, Mexico; ^4^ Neurology and Neurosurgery Center, Médica Sur Hospital, Mexico City, Mexico; ^5^ Departament of Neurophysiology, Universidad Autónoma de San Luis Potosí, San Luis Potosi, Mexico; ^6^ Anesthesia Department, Critical Care, and Pain Medicine, Beth Israel Deaconess Medical Center, Harvard Medical School, Boston, MA, United States; ^7^ Neurology, Médica Sur Hospital, Mexico City, Mexico; ^8^ Department of Neurology, General Hospital 450 Durango, Mexico City, Mexico; ^9^ Star Medica Group, Star Medica Hospital Lomas Verdes, Mexico City, Mexico; ^10^ Department of Neurology and Psychiatry, National Institute of Medical Science and Nutrition “Salvador Zubirán,” Mexico City, Mexico; ^11^ Department of Neurology, General Hospital of Culiacan, Culiacan, Mexico; ^12^ Headache and Chronic Pain Clinic, Neurology Service, Hospital Universitario “Dr. J. E. González” of the Universidad Autónoma de Nuevo Leon, Monterrey, Mexico; ^13^ Neurological Center, ABC Medical Center, Mexico City, Mexico; ^14^ Department of Neurology and Psychiatry, Clínica de Merida, Merida, Mexico City, Mexico; ^15^ Epilepsy Clinic of the National Institute of Neurology and Neurosurgery Manuel Velazco Suarez, Mexico City, Mexico

**Keywords:** cluster headache, treatment, clinical trials, drugs, neuromodulation

## Abstract

Cluster headache (CH) is the most common and devastating autonomic headache with multiple and recent advances in treatment. However, it usually goes unrecognized and is found to have a delayed and inappropriate treatment. This paper aims to review the current therapeutic options for patients with CH. We conducted a narrative literature review on the treatments available for this condition using the American Academy of Neurology (AAN) classification of therapeutic evidence. We found effective and safe pharmacological and non-pharmacological therapies with heterogeneity of clinical trial designs for patients with CH, and they are divided into three phases, namely, transitional, acute, and preventive interventions. Prednisone (A) is the most studied treatment in the transitional phase; acute attacks are treated using triptans (A), oxygen (A), and non-invasive transcutaneous vagal nerve stimulation (A). Verapamil (A) and monoclonal antibodies (possible A) are considered the first options in preventive treatments, followed by multiple pharmacological and non-pharmacological options in prophylactic treatments. In conclusion, numerous effective and safe treatments are available in treating patients with episodic, chronic, and pharmacoresistant CH according to the clinical profile of each patient.

## Introduction

Cluster headache (CH) is one of the most common primary headaches affecting 0.1% of the population (1). Unfortunately, an average delay of the diagnosis of CH is reported to be 5 years, and a minority of the patients do not receive adequate treatment (2). Multiple treatments for CH are currently available and can be used in acute, transitional, and preventive phases. In this study, we aim to perform a narrative review of treatment options for CH.

This paper is a narrative description of the state-of-the-art treatment of CH developed by a group of members of the Mexican Association of Headache and Migraine (AMCEMIG) from Mexico. A comprehensive research was conducted using the following databases: PubMed, PsycINFO, and Web of Science. The search terms included cluster headache treatment and “oxygen therapy,” triptans,” “acute,” “transcutaneous vagal nerve stimulation,” “sphenopalatine ganglion radiofrequency,” “sphenopalatine ganglion stimulation,” “prednisone,” “neuromodulation,” “cranial nerve infiltrations,” “preventive,” “verapamil,” “galcanezumab,” “lithium carbonate,” “gabapentin,” botulinum toxin,” “civamide,” “topiramate,” “baclofen,” “melatonin,” or “sodium valproate.” This search was limited to papers published in Spanish or English between 1960 and 2022.

### Selection criteria

Publications were included if they examined the effectiveness of treatments for CHs, including abortive and preventive therapies; case reports or case series with less than 10 patients were excluded. In total, 75 studies met the inclusion criteria.

All scientific articles were evaluated using the American Academy of Neurology (AAN) classification of evidence. This classification system considers study design, evidence quality, and consistency of results. Evidence is classified into four categories: Class I (strong evidence), Class II (moderate evidence), Class III (weak evidence), and Class IV (contradictory or insufficient evidence). The AAN classification includes five levels of evidence, as follows (Table 1):

1. Level A: Established as effective, ineffective, or harmful based on at least two Class I studies (randomized and controlled trials) or a systematic review of Class I studies.
2. Level B: Probably effective, ineffective, or harmful based on at least one Class I study, two or more Class II studies (non-randomized and controlled trials), or a systematic review of Class II studies.
3. Level C: Possibly effective, ineffective, or harmful based on at least one Class II study, two or more Class III studies (observational studies), or a systematic review of Class III studies.
4. Level U: Inadequate or contradictory data requiring additional studies.
5. Good practice: Based on expert consensus, standard practice, opinion, or case series for diagnostic, treatment, prevention, or screening studies.

**Table 1:** Levels of evidence according to the American Academy of Neurology of the use of several treatments in acute, transitional, and preventive stages.

Intervention in cluster headache	Level of evidence
Acute treatment	
Oxygen therapy	A
Triptans	A
Transcutaneous vagal nerve stimulation	A
Sphenopalatine ganglion stimulation	B
Sphenopalatine ganglion radiofrequency	C
Transitional treatment	
Prednisone	B
Cranial nerve infiltrations	B
Triptans	C
Transcutaneous vagal nerve stimulation	B
Sphenopalatine ganglion stimulation	U
Preventive treatment	
Verapamil	A
Galcanezumab	A
Lithium carbonate	B
Civamide	B
Transcutaneous vagal nerve stimulation	B
Botulinum toxin	C
Gabapentin	C
Topiramate	C
Baclofen	C
Sodium valproate	C
Melatonin	U

### Data extraction

Two independent reviewers for each intervention extracted data from each study using a standardized data form. The form included information on the study design, sample size, treatment type, treatment duration, primary outcome measure, and key findings. When there were any discrepancies between the two reviewers, they resolved them through discussion.

### Data synthesis

Due to the heterogeneity of the studies, a narrative synthesis approach was used to summarize the findings. The studies were grouped according to the treatment type, and the key findings were summarized in a narrative format. The strengths and limitations of each study were also discussed in several meetings and assigned a level of evidence according to the AAN therapeutic guidelines.

#### Acute treatment

**Figure 1:**
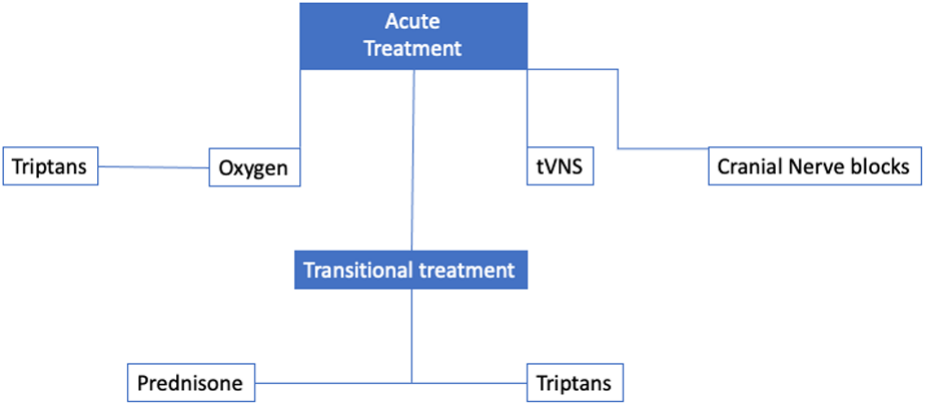
The acute and transitional treatment for CH.

This phase is treated with triptans, high-flow oxygen, and transcutaneous vagal nerve stimulation (tVNS) (3). Figure 1 shows the acute and transitional treatment for CH.

##### Triptans

Triptans with sufficient evidence in CH are subcutaneous (SC) sumatriptan, oral sumatriptan, and intranasal (IN) zolmitriptan.

SC sumatriptan (6 mg) is considered the gold standard to treat acute problems and is an effective drug in stopping a pain crisis in CH (4), in a controlled trial (*n* = 39), randomized to receive 6 mg SC sumatriptan vs. placebo. Sumatriptan was more effective than placebo in achieving 46% of patients being pain-free within ≤15 min compared with only 10% on placebo (*p* < 0.001) (5). In another randomized, double-blind study using 6 or 12 mg SC, mild to complete improvement within 15 min was achieved in 35% on placebo, 75% on 6 mg of SC sumatriptan, and 80% on 12 mg of SC sumatriptan, with no significant difference between the two active groups (5), and according to the report of Gregor et al., lower doses (2–6 mg SC) have been successfully used (6). SC sumatriptan should be administered twice daily if the patient continues to present with pain. A patient with CH is unlikely to develop medication-overuse headache (7–9), tachyphylaxis, increased pain crisis, or delayed medication effect (10–12). However, Dousset et al. (13) have reported that overuse of this SC triptan can aggravate the evolution of the problem.

Although sumatriptan is generally safe to use and does not cause serious adverse events (AEs) (except in patients with coronary artery disease), prophylactic management is the most important, as daily use of sumatriptan may increase the frequency and intensity of attacks (14).

In a randomized, double-blind study of IN sumatriptan, 57% of attacks decreased in severity within 20 min after 20 mg IN sumatriptan, being twice as effective as placebo (11). In a randomized open-label study, they compared 6 mg SC sumatriptan with 20 mg IN sumatriptan to relieve pain attacks within 5 min of pain onset. Of the 52 SC doses applied, 49 achieved complete relief within 15 min compared with seven out of 52 with IN treatments. Only two out of 26 patients preferred IN treatment (12).

Intranasal zolmitriptan 5 mg or 10 mg are similar, being more effective than oral zolmitriptan (15). In two randomized, double-blind studies, IN zolmitriptan was superior to placebo in reducing pain within 30 min after treatment by 38.5%–40% with 5 mg and 46.9%–62% with 10 mg (*p* < 0.001) (16). Slightly less than half of the patients on triptans presented with mild and transient adverse effects, such as paresthesia, chest pain, sore throat, and sensation of heat. Severe reported adverse events were transmural cardiac infarction, cardiac arrest, and arrhythmias, and caution should be exercised in patients with a cardiovascular history (17–22) (*level of evidence B*). However, a recent retrospective cohort including 130,000 migraine sufferers exposed to triptans vs. the same number of non-exposed individuals did not find an association between triptan use and increased cardiovascular risk (21) (*level of evidence B*). The US Food Drug Administration (FDA) recommends avoiding triptans in cardiac patients (22), limiting its clinical use (23).

In summary, 6 mg SC sumatriptan or 20 mg IN sumatriptan and 5/10 mg IN zolmitriptan are recommended for treating acute CH attacks (*level of evidence A*). They are not recommended for use in patients with cardiovascular risk factors (*level of evidence B*).

##### Oxygen therapy

This therapy has shown efficacy and safety in different studies (24). Since Horton's first trial in 1952 (25), 100% oxygen has been used for CH. In 1981, in an open-label randomized crossover trial, 52 patients with CH received 100% oxygen at 7 L/min through a simple face mask, achieving complete or near-complete pain relief in 62% of patients after 7 min, particularly in episodic CH (26). In the first randomized, crossover, double-blind, placebo trial, 19 patients with CH received oxygen inhaled at 6 L/min through a mask without re-inhalation for 15 min or compressed air as a placebo (27). Of the 16 patients on oxygen, 56% experienced complete relief or achieved substantial relief from the attack, compared with 7% of patients who inhaled air (27). In another recent study, 109 patients with CH were randomly assigned to use 100% high-flow oxygen inhaled at 12 L/min through a mask without re-inhalation for 15 min compared with a control group that inhaled air. High-flow oxygen was effective in aborting crisis in 78%, and freedom from pain was achieved at 15 min compared with only 20% of attacks treated with inhaled air (*p* < 0.001) (28).

An internet survey of 2,193 patients (of which 1,604 had CH and 504 had probable CH) demonstrated an excellent response to O_2_ treatment in almost 50% of patients. The efficacy was even higher in those over 65 (77%) (29). In a review, oxygen devices for CH were compared, and while it is true that the inspired fraction of O_2_ is better with high-flow devices, no significant difference in therapeutic outcomes was found (30). In a single-blind, semi-randomized, crossover-controlled study, 102 acute attacks were treated with 100% oxygen in 57 patients with CH using a simple mask at 15 L/min, valve mask for O_2_ on demand, or the O_2_ptimask, and no significant difference in efficacy was found. However, a *post hoc* analysis favored using the O_2_ on-demand valve mask and the O_2_ptimask by avoiding another rescue therapy (31).

In a randomized, double-blind crossover study, patients were treated with O_2_ at 7 and 12 L/min, with no significant difference found between the two doses in terms of efficacy for the primary outcome of pain control nor in the incidence of adverse effects. Similarly, it was found that those who did not respond to a dose of 7 L/min will show no benefit at doses of 12 L/min, an odds ratio of being pain-free using 12 L/min of 0.73 [95% confidence interval (CI) 0.52–1.02] compared with 7 L/min (*p* = 0.061) (32).

Other forms of oxygen therapy exist, such as continuous positive airway pressure and hyperbaric chamber for CH. However, a systematic review of hyperbaric oxygen reported low quality in evidence (33).

The use of oxygen in treating a painful crisis in CH is recommended (*level of evidence A*), with no a significant difference between the types of oxygen devices utilized.

##### Transcutaneous vagal nerve stimulation

This treatment was used in refractory epilepsy, using a stimulator of the vagus nerve implanted, which modulates the trigeminal autonomic reflex and central connections in the nucleus of the solitary tract to the hypothalamus via the vagus nerve (34). Ten (48%) CH patients (11 chronic, eight episodic, 15 improved, and four were unchanged) improved, with a mean of 11 ± 1 min from the start of pacing. Ten (55%) patients reduced acute high-flow oxygen, and nine (48%) patients reduced triptan use by 48%. Prophylactic use of the device substantially reduced attack frequency from 4.5/24 h to 2.6/24 h (*p* < 0.0005) after treatment (35).

A prospective, open-label, randomized trial (PREVA) compared adjuvant prophylactic VNS (*n* = 48) with standard care alone [control (*n* = 49)]. Two weeks of baseline were followed by a 4-week randomized phase (standard care plus VNS vs. control) and a 4-week extension (standard care plus VNS). Primary endpoints were reduction in the mean number of CH attacks per week, response rate, use of abortive medications, and safety/tolerability. During the randomized phase, individuals in the intention-to-treat population treated with standard therapy plus tVNS (*n* = 45) had a more significant reduction than controls (*n* = 48) (−5.9 vs. −2.1) for a mean of 3.9 fewer attacks per week (95% CI 0.5–7.2; *p* = 0.02). Higher response rates of ≥50% were also observed with standard therapy plus tVNS [40% (18/45)] vs. controls [8.3% (4/48); *p* < 0.001] without serious adverse events (36).

One hundred and fifty subjects were enrolled in this study and randomized (1:1) to receive tVNS or sham treatment for ≤1 month (double-blind phase); completers were eligible to enter a 3-month open-label tVNS phase. The primary endpoint was the response rate. The initial population was 133 subjects, of which 60 were tVNS-treated subjects (episodic CH, *n* = 38; with chronic CH, *n* = 22) and 73 were sham-treated subjects (episodic CH, *n* = 47; chronic CH, *n* = 26). The response was achieved in 26.7% of tVNS-treated subjects and 15.1% of sham-treated subjects (*p* = .1). The rates of answer were significantly higher with tVNS than with sham (tVNS, 34.2%; sham, 10.6%; *p* = 0.008) but not in the chronic CH cohort (tVNS, 13.6%; sham, 23.1%; *p* = 0.48). Sustained response was higher with tVNS in the episodic CH (*p* = 0.008) and in the overall population (*p* = 0.04). No serious adverse events occurred (37). Goadsby et al. compared 48 subjects treated with tVNS (14 episodic CH, 34 chronic CH) and 44 subjects treated with sham (13 episodic CH, 31 chronic CH). For the primary endpoint, the treatments were not significantly different for the total cohort. In the episodic CH subgroup, tVNS (48%) was superior to sham (6%; *p* < 0.01). But neither in the chronic form (38).

In patients with drug-resistant CH or pharmacological contraindications, tVNS is indicated as acute or preventive treatment alone or in addition to the standard treatment (*level of evidence B*).

##### Treatments in the sphenopalatine ganglion

Radiofrequency (RF) blockade or stimulation with automatic devices of the sphenopalatine ganglion (SPG) has been used for refractory cases of CH.

##### Radiofrequency denervation

In 1997, Sanders and Zuurmond performed sphenopalatine ganglion blockade (*n* = 66) using a fluoroscopy-guided procedure and RF ablation of the sphenopalatine ganglion for the management of CH, achieving complete pain relief in 60.7% of patients with episodic CH and 30% of patients with chronic CH with few transient side effects (39).

In a recent case series on the effect of RF denervation of the sphenopalatine ganglion in 23 patients with drug-resistant CH, 79% experienced more than 50% pain relief at the first procedure, and after undergoing denervation, the pain relief was 77% at 48 h, and 59%, 60%, and 31% at 1, 3, and 6 months, respectively, concluding that this treatment can effectively decrease CH pain for at least several months (40) (*level of evidence C*).

##### Sphenopalatine ganglion stimulation

In the first randomized controlled trial of 28 patients with chronic CH, 566 attacks were treated using a remotely controlled device designed to stimulate the sphenopalatine ganglion through a device implanted in the pterygopalatine fossa on the side of the headache. At 15 min of sphenopalatine ganglion stimulation, pain relief was obtained in 67.1% of attacks treated with complete stimulation vs. 7.4% with sham stimulation, and a significant absence of pain was achieved in 34.1% vs. 1.5%. The acute response rate was 32%. Adverse events of maxillofacial surgery were frequent but reversible. Eighty-one percent of the patients experienced maxillary paresthesia, and six patients were re-operated (40). In their open phase, 32 patients with pain relief in 67.1% at 15 min were successfully relieved by neuromodulation, while freedom from pain was obtained in only 7.4% with sham stimulation (*p* < 0.0001) (41). In an evaluation of 5,956 attacks of pain over 24 months, 45% responded in patients with chronic CH refractory to SPG stimulation. This effective acute treatment offers sustained efficacy over 24 months of observation (42). In a population of 33 patients with refractory chronic CH, SPG neuromodulation induced periods of cluster attack remission; some patients were also able to reduce or discontinue their preventive medication, and remissions were accompanied by improved quality of life (43).

CH with RF ablation has a utility *level of evidence C*. Treatment with different remotely controlled devices for sphenopalatine ganglion stimulation has *evidence B*.

##### Cranial nerve infiltrations

Few studies showed effectiveness for complete attack control of 35.6% and partial response of 64.4%. Peres et al. proposed the use of the block of the great occipital nerve as a transitional treatment for the attack of CH in the acute presentation, with good tolerance and no adverse events; headache intensity, frequency, and duration significantly decreased compared with the week before and the week after the nerve block (*p* < 0.003, *p* = 0.003, *p* < 0.005, respectively) (44). An open-label, uncontrolled study of multiple pericranial nerve infiltrations showed effectiveness using major occipital nerve infiltration for chronic CH, and this therapy can be more effective or inclusive than prednisone in the transitional treatment (82.7% vs. 64.4%) (45).

#### Transitional treatment: short prevention or bridge

To treat a cluster attack, to serve as a temporary “bridge” between acute and preventive treatments. Its use depends on initiating a preventive treatment or the daily frequency of attacks. It is generally recommended during an outbreak (cluster) with ≥2 daily attacks. Also, it has been proposed that the history of the duration of each episode should be greater than or equal to the latency for the effect of long-term preventive treatments. Scientific evidence for the transitional treatment is limited (45). Multiple cranial nerve blocks may provide an effective, well-tolerated, reproducible transitional treatment in CH (46).

##### Prednisone

Prednisone is a drug effective for the treatment of CH with only limited high-quality evidence. Only one prospective, randomized, placebo-controlled study (53 vs. 56 placebo) was reported, using 100 mg prednisone for 5 days and tapering 20 mg every 3 days and simultaneously starting verapamil at 40 mg TID and increasing to 120 mg TID by day 19 at the beginning of the treatment and ending with a final dose of 360 mg/day. Patients in the prednisone group had a mean of 7-1 (SD 6-5) attacks in the first week, compared with 9-5 (6-0) attacks in the placebo group of patients (*p* = 0.002) (47). Therefore, it is recommended to use prednisone (100 mg/day) for 5 days and taper 20 mg every 3 days until finished (*level of evidence B*)***.***

##### Triptans

Any recent high-quality studies on the effectiveness of triptans in transitional treatments are yet to be reported. A case series with naratriptan [in one report, 43 cases, 37 patients (86.0%) achieved an improvement of CH] (48) and frovatriptan can be effective as a transitional therapy for CHs (49). A small open-label study with 18 patients suggests that 40 mg eletriptan, administrated twice daily, may be helpful for transition prophylaxis in CH (50) (*level of evidence III C*).

In a 2005 study (51), 11 out of 13 (85%) patients in the active group (of which four had chronic CH and nine had episodic CH) were attack-free compared with 0 out of 10 patients in the placebo group (*p* = 0.0001), with preparations as follows: propionate and disodium phosphate, plus lidocaine, into the greater occipital nerve ipsilateral to the pain, vs. lidocaine alone, reporting that 85% of patients were attack-free at 72 h vs. none in the placebo group and 30.7% had immediate response. No significant difference was found if the patient was previously receiving preventive treatment (51).

In another similar study, a suboccipital injection of cortivazol was performed in patients with episodic or chronic CH. The protocol included an administration of three injections of the drug, without local anesthetic, over 6 days, with evaluation at 9, 30, and 90 days and up to 11 months (52). Forty-three patients were enrolled in the study. Results favored cortivazol, and 52% of the patients remained pain-free from day 4 to 30, with a mean difference of 19.7, 6.8–32.6; *p* = 0.004) (52) (*level of evidence B*).

##### Neuromodulation

No specific studies for transitional treatment in CH were reported. However, studies of sphenopalatine ganglion stimulation and percutaneous vagal nerve stimulation showed indirect evidence, and in the former case, it was not recommended (41).

###### Non-invasive transcutaneous vagal stimulation

A prospective randomized open-label study evaluated this technique for the acute management of refractory chronic CH (42), using SPG stimulation for disabled patients, and should be considered after medical treatments fail (*level of evidence B*).

Anti-CGRP monoclonal antibodies were not approved as bridge therapy and will be discussed in the next section (53).

#### Preventive treatment

##### Verapamil

**Figure 2:**
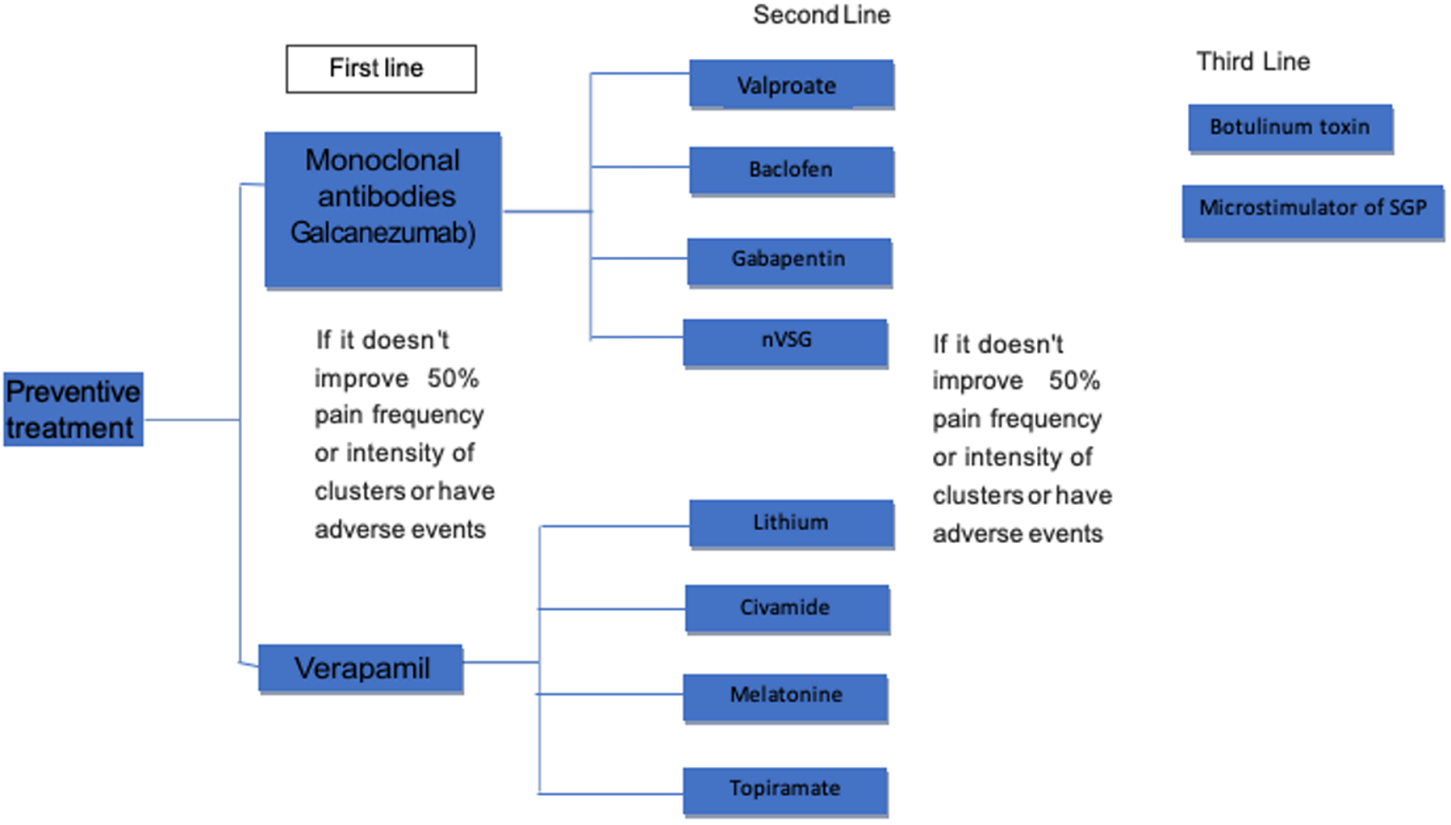
The preventive treatment algorithm.

Figure 2 shows the preventive treatment algorithm. This drug is the preventive drug of first choice for CH (54). Only five trials (two controlled) have investigated its prophylactic effect. In a double-blind study comparing the efficacy of verapamil vs. placebo in episodic CH prophylaxis, in 30 patients, 15 on verapamil (120 mg TID) and 15 on placebo (TID) for 14 days, 360 mg verapamil vs. placebo showed a significant decrease in daily attack frequency (0.66 ± 0.8.8 vs. 1.65 ± 1.01, respectively, *p* < 0.0001) and everyday analgesic use (0.5 ± 0.87 vs. 1.2 ± 1.03, respectively, *p* < 0.004). They are reporting a reduced frequency of attacks and abortifacient consumption in the verapamil group. Side effects were mild (55).

In a report of 369 patients with CH, 217 outpatients (175 men) received verapamil, starting at a minimum of 240 mg/day and increasing by 80 mg every 2 weeks with a control electrocardiogram (EKG), until the CH stopped, or side effects prevented increasing the dose, or until a maximum dose of 960 mg/day was reached. However, one patient received verapamil of 1,200 mg/day. In total, 19% had arrhythmias, and 36% had bradycardia. The authors suggested performing EKGs and monitoring for the development of block and bradycardia in any patient receiving verapamil (56).

In a multicenter trial with a double-blind, crossover design, comparing verapamil (360 mg/day) with lithium carbonate (900 mg/day) in preventing crisis in chronic CH, both effectively prevented CH. However, verapamil caused fewer side effects and had a shorter period of response latency. No correlation was observed between the plasma levels of the drugs and their clinical efficacy.

Both significantly improved the headache index (verapamil 50%, lithium 37%) and the reduction in analgesic consumption (58% in both groups) (57).

An open trial showed that 94% (49/52) of patients with episodic CH and 55% (10/18) with chronic CH improved with doses of 200–960 mg. However, two patients with episodic CH and eight with chronic CH required additional therapy (lithium, sumatriptan, or valproate) (58). In another open clinical trial (*n* = 48) regarding CH, 69% improved at ≥75%, finding no difference in response between episodic (mean dose of 354 mg/day) and chronic (mean dose of 572 mg/day) CH (59).

In the current daily practice, patients receive verapamil at an average dose of 578 mg daily (maximum of 1,200 mg daily). The dose should be increased slowly to minimize side effects and to determine the lowest effective dose. Adverse effects are mainly cardiac conditions. In 2016, 22 cardiologists recommended that an EKG should always be performed before treatment, but no consensus on monitoring during treatment is agreed. Fifty percent recommended performing an EKG before dose escalation, while 60% recommended an EKG after dose escalation (60).

The European Federation of Neurological Societies (EFNS) guidelines for the treatment of CH suggest that prophylaxis should be performed with verapamil with a daily dose of 240 mg (maximum dose depends on tolerability or efficacy) (61). In several recent updates, the dose of 360 mg/day divided into three doses of 120 mg each is suggested (62).

In summary, in both episodic and chronic CHs, verapamil effectively reduces the intensity and number of pain attacks, facilitates acute treatment, and is the most robust evidence of maintenance preventive therapy (*level of evidence A*).

##### Anti-CGRP mAbs

Monoclonal antibodies that act by binding to CGRP, fremanezumab, and galcanezumab have been evaluated for the preventive treatment of CH. Galcanezumab at high doses (300 mg) is effective in the prevention of episodic CH. However, fremanezumab and galcanezumab at non-high doses are ineffective in preventing chronic CH (63). Galcanezumab was approved by the FDA; based on a study of 106 enrolled patients, 49 were randomized to galcanezumab and 57 to placebo, with a mean baseline of 17.8 ± 10.1 in the galcanezumab group and 17.3 ± 10.1 in the placebo group. The weekly reduction in attacks was 8.7 in the galcanezumab group compared with 5.2 in the placebo group (difference of 3.5 attacks weekly; 95% CI, 0.2–6.7; *p* = 0.04). The adverse effects were comparable with placebo (53). Recent data from randomized trials using monoclonal antibodies targeting CGRP (anti-CGRP mAbs) are controversial. Galcanezumab is the first anti-CGRP mAb approved by the FDA for episodic CH, although the study for chronic CH was negative (64). The administration is subcutaneous: 300 mg monthly. The most frequent side effects were nasopharyngitis and pain at the application site (65). In episodic CH, galcanezumab is a first-line treatment that decreases the intensity and number of pain attacks and facilitates preventive treatment. Two hundred thirty-seven patients were randomized and treated (120 with placebo; 117 with galcanezumab). The primary endpoint was the mean change from baseline in weekly attack frequency with galcanezumab compared with placebo. The secondary endpoints were a 50% response rate. At the start of the study, the age was 45 years, and 63% were taking a preventive drug. The primary endpoint was not reached; the mean change in weekly attack frequency was 4.6 with placebo. The mean change in weekly attack frequency was 4.6 with placebo vs. 5.4 with galcanezumab (*p* < 0.334) (66). In a recent study, 233 patients received at least one dose of galcanezumab over 341 days. Most were male (*n* = 169/233; 72.5%) with a mean age of 44.9 (±10.9) years. A total of 185 patients (*n* = 185/233; 79.4%) reported adverse events, and 18 patients (*n* = 18/233; 7.7%) discontinued treatment due to AEs. Probable hypersensitivity episodes (rash, urticaria, and injection site) (*n* = 14/233; 6.0%) were reported. A history of suicidal ideation (*n* = 55/237; 23.2%) was found, and one patient had a non-fatal suicide attempt during washout. Galcanezumab at 300 mg monthly had a good tolerability and safety profile in adults with chronic CH with up to 15 months of treatment (67). Therefore, this is a promising treatment, although at the moment, due to a lack of controlled clinical studies, more evidence for 1A recommendation for episodic CH is needed. Still, it may be at this level in the future.

##### Lithium carbonate

Lithium is used if verapamil fails or a verapamil treatment cannot be initiated or continued. Three trials with lithium are noted. In a preemptive multicenter study using a double-blind, crossover design, verapamil was compared with lithium carbonate, with both being effective. Still, verapamil caused fewer side effects and had a shorter latency period (68).

In a double-blind, placebo-controlled, parallel-group, comparative study evaluating slow-release lithium (800 mg/day) or placebo, lithium was discontinued in patient 27 because no difference was found with placebo. Only minor adverse events were reported (69). In a retrospective study of 19 patients, eight with chronic CH experienced a positive effect using lithium in the first 2 weeks (serum concentration of 0.7–1.2 mmol/L) (68). Studies suggested that lithium therapy is more effective in chronic than episodic CH, but further studies are needed (69).

Side effects may induce discontinuation of treatment. Nausea, dizziness, and tremor are some of the side effects of lithium. Prolonged use of lithium carbonate may cause renal failure and lead to hypothyroidism. To minimize side effects, serum concentrations and hepatic, thyroid, and renal function should be periodically monitored. Lithium concentration should not exceed 1.2 mEq/L. In CH prophylaxis, which is possibly more chronic than episodic, lithium is an effective treatment and could be used if first-line management (verapamil, maybe galcanezumab) has not worked (*level of evidence B*) (70).

##### Sodium valproate

In an open trial, sodium valproate (600–2,000 mg/day) effectively treated CH in 11 out of 15 (73%) patients, and nine achieved complete remission (71). Subsequently, a double-blind, placebo-controlled clinical study with 50 patients (of which 37 had CH) found no difference between the sodium valproate group and placebo group, with a 50% decrease in attack frequency in sodium valproate and 62% in placebo (72). Sodium valproate could be an alternative in preventing CH, although evidence of its efficacy is only limited (level of evidence C).

##### Civamide

A nasal spray (10 μl/0%–025%) is applied daily based on capsaicin that inhibits pain transmission by activating valinoid-1 receptors and blocking calcium channels. The most frequent side effects are nasal burning, tearing, and rhinorrhea. In a multicenter, double-blind, placebo-controlled study, a significant improvement in the frequency of pain attacks (−8.6 vs. 3.6) from week 1 to day 14 post-treatment was seen (73) without significant changes in intensity, severity of headaches, and associated symptoms.

In CH, nasal civamide only improves the frequency of pain attacks (*level of evidence B*).

##### Gabapentin

Gabapentin interacts with calcium channels and increases GABA synthesis in the central nervous system (74). In an open pilot study with 12 patients who did not respond to other treatments, gabapentin was effective in significantly reducing the frequency and intensity of headache as a prophylactic treatment for CH, observing that after 8 days, the pain had subsided, an effect that was maintained for up to 4 months (75).

In another study of 14 patients (with mean age of 42 ± 15 years), gabapentin was gradually introduced; the maintenance dose was 900–2,400 mg for an average of 3.5 months. The average number of headaches per week and pain intensity were reduced; only two (14.28%) patients did not respond. At the end of the treatment with gabapentin, no recurrences in the treated patients were noted. Mild to moderate side effects, such as drowsiness, dizziness, sluggishness, and constipation, were registered in 57.14%. No dropouts due to side events were reported (76).

In another open-label study, eight patients with episodic CH and four patients with chronic refractory CH received 900 mg/day of gabapentin, and all were pain-free at 1 week. In six patients with episodic CH, treatment was discontinued after 2 months, and no relapses occurred during 3 months of follow-up.

No new attacks were recorded during 4 months of follow-up in patients with chronic CH, and only two reported mild somnolence. Gabapentin also proved effective in patients with chronic CH; six out of eight patients who were resistant to a first-line treatment responded to this therapy. The most prolonged continuous pain-free period with a constant dose was up to 13 months. In another patient, an exacerbation of CH occurred; therefore, gabapentin was increased to 2,400 mg, and daily and oral corticosteroids were transiently added (10 days).

CH attacks almost or completely suppressed markedly vary (between 800 and 3,600 mg). Undesirable effects are transient somnolence with high doses of gabapentin and impotence (77). Gabapentin may be helpful in the prevention of both episodic and chronic CHs alone or in combination with other drugs (*level of evidence C*).

##### Topiramate

This drug is antiepileptic with multiple mechanisms of action. According to an open-label, prospective study of 12 patients with episodic CH and 14 patients with chronic CH, starting with 25 mg to 200 mg, a remission in 15 patients and six with more than 50% was noted. Seven patients had remission since the beginning. The most frequent side effects of topiramate are cognitive dysfunction, paresthesia, taste alterations, weight loss, fatigue, and dizziness. It is contraindicated in patients with a history of nephrolithiasis (78).

In episodic and chronic CHs, topiramate is recommended as a preventive option treatment (*level of evidence C*).

##### Baclofen

Baclofen acts on GABA-B receptor activation. In an open-label, prospective study with 16 symptomatic patients who suffered from CH, the patients received 15 mg to 30 mg for 2 weeks, and 12 patients were attack-free for 1 week using 5 mg–10 mg per day. The most common side effects of baclofen are dizziness, ataxia, muscle weakness, and drowsiness (79).

In episodic CH, baclofen is recommended as a preventive treatment (*level of evidence C*).

##### Botulinum toxin

A systematic review on botulinum toxin (BTX) for the treatment of CH analyzed three studies (10–17 patients each), all showing a significant improvement in reducing the frequency and severity of CH by at least 50%, even in the first week of the treatment. SPG injection was shown to have a higher incidence of adverse events (80). Another pilot study showed that BTX in the otic ganglion did not reduce the number of attacks per week at 2 months (81). Ultrasound-guided large occipital nerve block using BTX has been used in nine subjects with CH, with a satisfactory response, and has been proposed as a treatment possibility (82).

In summary, BTX applied over the greater occipital nerve or in the sphenopalatine ganglion may be helpful for prophylaxis of both episodic and chronic CHs, alone or combined with other drugs (*level of evidence C*). A fascinating long-term study using the application of stereotactically guided BTX to the SPG in seven subjects showed a significant long-term reduction in the number of seizures in CH patients who received repeated injections. A considerable reduction was demonstrated in the total number of CH attacks and severe and unbearable symptoms, with a significant increase in headache-free days. This new technique uses a stereotactic lateral percutaneous approach to inject BTX into the SPG. The disadvantage is that it requires continuous x-ray imaging (fluoroscopy) and detailed anatomical knowledge of the area; the procedure can be repeated as often as necessary using a single CT image. This process is also well accepted and safe. However, a randomized, placebo-controlled trial is required to confirm its efficacy and safety (83). In a prospective, open-label, uncontrolled pilot study, a single injection of 25 IU (*n* = 5) or 50 IU (*n* = 5) BTA was administered into the SPG to 10 individuals with intractable chronic CH with a follow-up of 24 weeks. A total of 11 AEs were recorded to be non-severe; the most relevant was a posterior epistaxis. The number of attacks was significantly reduced from baseline from 11 ± 14 (*p* = 0.038) to 5 ± 5 (*p* = 0.028) at month 1; for months 2–6, the mean values and significance levels were identical to those of the analysis. The mean reduction of attacks from baseline in months 1–3 and months 4–6 for the entire follow-up (months 1–6) was 51% (*p* = 0.028). The frequency of cluster attacks was significantly reduced during 5 of the 6 months post-treatment. BTA injection into the SPG in intractable chronic CH appeared to have an acceptable AE profile. Efficacy data indicated a significant reduction in the frequency of the cluster attack after treatment, and five out of 10 patients responded to the treatment with a mean decrease in attack frequency of 77%. Randomized placebo-controlled studies are therefore warranted to establish the safety and efficacy of this treatment (84).

##### Occipital nerve stimulation

Since Magis et al. reported that for drug-resistant chronic CH, occipital nerve stimulation (ONS) may be helpful, several studies have been performed; in this authors’ analysis, eight patients with drug-resistant CH had a suboccipital neurostimulator implanted on the suboccipital side of the head and were asked to record details of the frequency, intensity, and symptomatic treatment of their attacks. Eight patients had a suboccipital neurostimulator implanted on the headache side and recorded the frequency, intensity, and symptomatic treatment of their attacks in a diary before and after the stimulator. Two patients were pain-free. Three patients had a reduction in seizure frequency of about 90%. Two patients had an improvement of about 40%. The mean follow-up was 15.1 months (SD 9.5, range 3–22). The procedure acts through slow neuromodulatory processes at the level of the upper brainstem or diencephalic centers (85).

Other open-label studies support “ONS” as a valuable tool in treating patients with refractory chronic CH. The potential side effects and complication rates of the intervention are minimal (86).

The efficacy of ONS was evaluated in an open-label study including 35 patients with chronic drug-resistant CH (with mean age of 42 years; 30 men; with mean illness duration of 6.7 years). The primary endpoint was a reduction in the number of daily attacks. After a median follow-up of 6.1 years (range 1.6–10.7), out of 20 (66.7%) responders (≥50% reduction in headache number per day), 12 (40%) showed a stable condition with sporadic attacks, five had a 60%–80% reduction in the number of headache attacks per day, and in the remaining three responders, chronic CH was transformed into episodic CH. Ten (33.3%) patients were non-responders. The efficacy of ONS is confirmed in chronic drug-resistant CH (87) (*level of evidence B*).

##### Melatonin

Small, non-randomized clinical studies have not demonstrated that melatonin is used to prevent CH (88, 89).

In conclusion, melatonin is not effective in the preventive treatment of CH (*level of evidence U*).

## Conclusions

Multiple effective and safe treatments are available to treat patients with episodic, chronic, and pharmacoresistant CH according to the profile of each patient.

Choosing the best treatment in an individualized and appropriate way can change the quality of life of those who suffer from a terrible condition, which, due to its severity, has been called the “suicidal headache” because of the anxiety caused by acute, episodic, or chronic headaches.
